# 3,5,3′,5′-Tetra­methyl-4,4′-bi(1*H*-pyrazol­yl) hemihydrate

**DOI:** 10.1107/S1600536812037920

**Published:** 2012-09-08

**Authors:** Shouwen Jin, Yanfei Huang, Hao Fang, Tianyi Wang, Liangliang Ding

**Affiliations:** a Tianmu College of ZheJiang A & F University, Lin’An 311300, People’s Republic of China

## Abstract

In the title compound, C_10_H_14_N_4_·0.5H_2_O, the amino H atom of one of the two pyrazole rings is disordered over its two N atoms in a 1:1 ratio. The pyrazole rings are aligned at 60.1 (1)°. In the crystal, two bipyrazolyl mol­ecules are linked by an N—H⋯N hydrogen bond, generating a dimer; the dimer is connected to the water mol­ecule, which lies on a twofold rotation axis, resulting in the formation of a chain that makes an angle of *ca* 45.3 (1)° with the *ab* plane. The chains are cross-linked by N—H⋯O and O—H⋯N inter­actions, forming a three-dimensional network.

## Related literature

For general background to coordination compounds based on 3,5,3′,5′-tetra­methyl-1*H*,1′*H*-[4,4′]bipyrazolyl, see: Boldog *et al.* (2001 ▶); Zhang & Kitagawa (2008 ▶). 
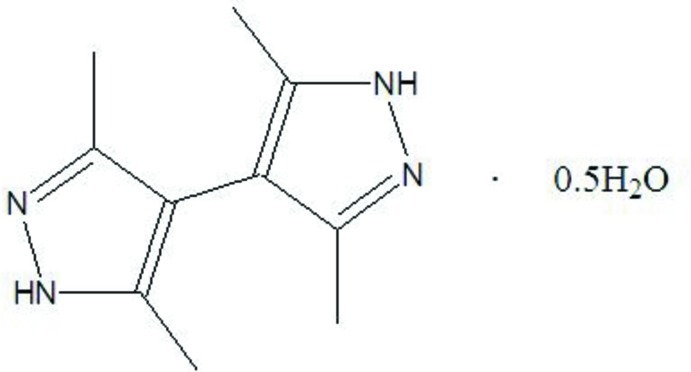


## Experimental

### 

#### Crystal data

C_10_H_14_N_4_·0.5H_2_O
*M* *_r_* = 199.26
Tetragonal, 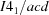

*a* = 24.9060 (4) Å
*c* = 14.9684 (2) Å
*V* = 9285.0 (2) Å^3^
*Z* = 32
Mo *K*α radiation
μ = 0.08 mm^−1^
*T* = 293 K
0.40 × 0.38 × 0.37 mm

#### Data collection

Bruker SMART 1K CCD area-detector diffractometer
Absorption correction: multi-scan (*SADABS*; Bruker, 2002 ▶) *T*_min_ = 0.970, *T*_max_ = 0.973
27495 measured reflections
2050 independent reflections
1480 reflections with *I* > 2σ(*I*)
*R*_int_ = 0.070

#### Refinement

*R*[*F* ^2^ > 2σ(*F* ^2^)] = 0.053
*wR*(*F* ^2^) = 0.169
*S* = 1.10
2050 reflections
144 parameters
1 restraint
H atoms treated by a mixture of independent and constrained refinement
Δρ_max_ = 0.23 e Å^−3^
Δρ_min_ = −0.15 e Å^−3^

### 

Data collection: *SMART* (Bruker, 2002 ▶); cell refinement: *SAINT* (Bruker, 2002 ▶); data reduction: *SAINT*; program(s) used to solve structure: *SHELXS97* (Sheldrick, 2008 ▶); program(s) used to refine structure: *SHELXL97* (Sheldrick, 2008 ▶); molecular graphics: *SHELXTL* (Sheldrick, 2008 ▶); software used to prepare material for publication: *SHELXTL*.

## Supplementary Material

Crystal structure: contains datablock(s) global, I. DOI: 10.1107/S1600536812037920/ng5290sup1.cif

Structure factors: contains datablock(s) I. DOI: 10.1107/S1600536812037920/ng5290Isup2.hkl

Supplementary material file. DOI: 10.1107/S1600536812037920/ng5290Isup3.cml

Additional supplementary materials:  crystallographic information; 3D view; checkCIF report

## Figures and Tables

**Table 1:** Hydrogen-bond geometry (Å, °)

*D*—H⋯*A*	*D*—H	H⋯*A*	*D*⋯*A*	*D*—H⋯*A*
O1—H1*D*⋯N4	0.84 (1)	1.95 (1)	2.791 (2)	175 (2)
N3—H3⋯O1^i^	0.88 (3)	1.96 (3)	2.827 (2)	169 (2)
N2—H2⋯N2^ii^	0.91 (5)	2.23 (5)	3.015 (4)	144 (5)
N1—H1⋯N1^iii^	0.91 (5)	1.98 (5)	2.862 (4)	164 (5)

Symmetry codes: (i) 
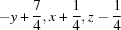
; (ii) 
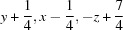
; (iii) 
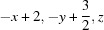
.

**Fig. 1.**
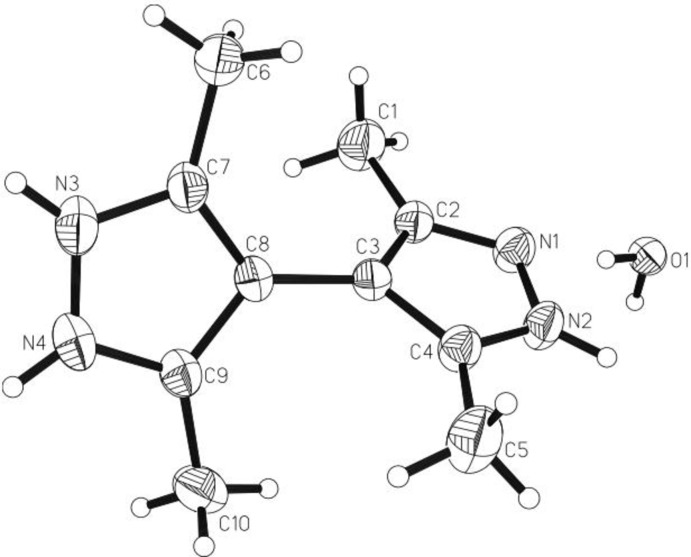
The structure of the title compound, showing the atom-numbering scheme. Displacement ellipsoids are drawn at the 30% probability level.

**Fig. 2.**
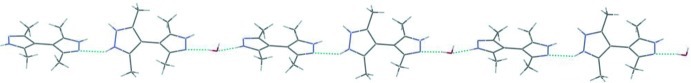
The chain structure formed through the nonbonding interactions.

## supplementary crystallographic information

### Comment

The use of 3,5,3',5'-tetramethyl-1H,1'H-[4,4']bipyrazolyl has drawn strong interest in coordination chemistry (Zhang *et al.*, 2008, Boldog *et al.*, 2001).

In the crystal structure of C_10_H_14_N_4_^.^0.5H_2_O, the amino H atom of one of the two pyrazole rings is disordered over its two N atoms in a 1:1 ratio. The two pyrazole rings are aligned at 60.1 (1)°. Two C_10_H_14_N_4_ molecules are linked by an N–H···N hydrogen bond to generate a dimer; the dimer is connected to the water molecule, which lies on a twofold rotation axis to chain that makes an angle of *ca* 45.3 (1)° with the *ab* plane. The chains were crosslinked together by N—H···O, and O—H···N associations to form a three-dimensional network structure

### Experimental

3,5,3',5'-Tetramethyl-1*H*,1'*H*-[4,4']bipyrazolyl was prepared according to the literature and crystals were grown from its solution in ethanol. (Boldog *et al.* 2001).

### Refinement

H atoms bonded to N, and O atoms were located in a difference Fourier map and refined isotropically.

Other H atoms were positioned geometrically with C—H = 0.96 Å, and constrained to ride on their parent atoms with *U*_iso_(H) = 1.2Ueq(C).

### Crystal data

**Table:**

C_10_H_14_N_4_·0.5H_2_O	*D*_x_ = 1.140 Mg m^−^^3^
*M**_r_* = 199.26	Mo *K*α radiation, λ = 0.71073 Å
Tetragonal, *I*4_1_/*a**c**d*	Cell parameters from 3432 reflections
Hall symbol: -I 4bd 2c	θ = 2.6–28.4°
*a* = 24.9060 (4) Å	µ = 0.08 mm^−^^1^
*c* = 14.9684 (2) Å	*T* = 293 K
*V* = 9285.0 (2) Å^3^	Block, colorless
*Z* = 32	0.40 × 0.38 × 0.37 mm
*F*(000) = 3424	

### Data collection

**Table:**

Bruker SMART 1K CCD area-detector diffractometer	2050 independent reflections
Radiation source: fine-focus sealed tube	1480 reflections with *I* > 2σ(*I*)
Graphite monochromator	*R*_int_ = 0.070
φ and ω scans	θ_max_ = 25.0°, θ_min_ = 3.0°
Absorption correction: multi-scan (*SADABS*; Bruker, 2002)	*h* = −29→29
*T*_min_ = 0.970, *T*_max_ = 0.973	*k* = −24→29
27495 measured reflections	*l* = −17→16

### Refinement

**Table:**

Refinement on *F*^2^	Primary atom site location: structure-invariant direct methods
Least-squares matrix: full	Secondary atom site location: difference Fourier map
*R*[*F*^2^ > 2σ(*F*^2^)] = 0.053	Hydrogen site location: inferred from neighbouring sites
*wR*(*F*^2^) = 0.169	H atoms treated by a mixture of independent and constrained refinement
*S* = 1.10	*w* = 1/[σ^2^(*F*_o_^2^) + (0.0974*P*)^2^ + 1.819*P*] where *P* = (*F*_o_^2^ + 2*F*_c_^2^)/3
2050 reflections	(Δ/σ)_max_ = 0.001
144 parameters	Δρ_max_ = 0.23 e Å^−^^3^
1 restraint	Δρ_min_ = −0.15 e Å^−^^3^

### Special details

**Table:**

Geometry. All e.s.d.'s (except the e.s.d. in the dihedral angle between two l.s. planes) are estimated using the full covariance matrix. The cell e.s.d.'s are taken into account individually in the estimation of e.s.d.'s in distances, angles and torsion angles; correlations between e.s.d.'s in cell parameters are only used when they are defined by crystal symmetry. An approximate (isotropic) treatment of cell e.s.d.'s is used for estimating e.s.d.'s involving l.s. planes.
Refinement. Refinement of *F*^2^ against ALL reflections. The weighted *R*-factor *wR* and goodness of fit *S* are based on *F*^2^, conventional *R*-factors *R* are based on *F*, with *F* set to zero for negative *F*^2^. The threshold expression of *F*^2^ > σ(*F*^2^) is used only for calculating *R*-factors(gt) *etc*. and is not relevant to the choice of reflections for refinement. *R*-factors based on *F*^2^ are statistically about twice as large as those based on *F*, and *R*- factors based on ALL data will be even larger.

### Fractional atomic coordinates and isotropic or equivalent isotropic displacement parameters (Å^2^)

**Table:**

	*x*	*y*	*z*	*U*_iso_*/*U*_eq_	Occ. (<1)
N1	0.94307 (8)	0.75779 (9)	0.78322 (15)	0.0541 (6)	
H1	0.9776 (18)	0.747 (2)	0.788 (3)	0.065*	0.50
N2	0.90490 (9)	0.72523 (9)	0.81759 (15)	0.0553 (6)	
H2	0.915 (2)	0.694 (2)	0.843 (4)	0.066*	0.50
N3	0.77810 (8)	0.89553 (8)	0.65442 (13)	0.0456 (5)	
H3	0.7666 (10)	0.9167 (10)	0.6112 (17)	0.055*	
N4	0.76044 (8)	0.90310 (8)	0.73926 (12)	0.0466 (5)	
O1	0.70458 (8)	1.0000	0.7500	0.0384 (5)	
H1D	0.7224 (8)	0.9711 (6)	0.7496 (15)	0.046*	
C1	0.95234 (11)	0.84574 (12)	0.7094 (2)	0.0661 (8)	
H1A	0.9842	0.8510	0.7444	0.099*	
H1B	0.9317	0.8783	0.7087	0.099*	
H1C	0.9621	0.8363	0.6495	0.099*	
C2	0.91976 (9)	0.80188 (9)	0.74928 (16)	0.0437 (6)	
C3	0.86404 (8)	0.79798 (9)	0.76267 (14)	0.0371 (5)	
C4	0.85697 (9)	0.74878 (10)	0.80591 (15)	0.0448 (6)	
C5	0.80618 (11)	0.72243 (11)	0.8364 (2)	0.0743 (9)	
H5A	0.8099	0.6842	0.8321	0.111*	
H5B	0.7769	0.7341	0.7995	0.111*	
H5C	0.7992	0.7322	0.8974	0.111*	
C6	0.84046 (14)	0.84292 (14)	0.56201 (18)	0.0778 (10)	
H6A	0.8343	0.8711	0.5195	0.117*	
H6B	0.8246	0.8102	0.5406	0.117*	
H6C	0.8784	0.8379	0.5697	0.117*	
C7	0.81576 (9)	0.85775 (9)	0.64938 (14)	0.0411 (6)	
C8	0.82394 (8)	0.83821 (8)	0.73500 (14)	0.0360 (5)	
C9	0.78832 (9)	0.86804 (9)	0.78872 (15)	0.0414 (6)	
C10	0.77949 (12)	0.86516 (12)	0.88733 (17)	0.0670 (8)	
H10A	0.7599	0.8962	0.9066	0.100*	
H10B	0.8135	0.8640	0.9173	0.100*	
H10C	0.7594	0.8334	0.9014	0.100*	

### Atomic displacement parameters (Å^2^)

**Table:**

	*U*^11^	*U*^22^	*U*^33^	*U*^12^	*U*^13^	*U*^23^
N1	0.0405 (12)	0.0563 (14)	0.0656 (14)	0.0158 (11)	−0.0071 (10)	−0.0029 (11)
N2	0.0478 (13)	0.0522 (14)	0.0659 (15)	0.0120 (11)	−0.0155 (10)	0.0047 (11)
N3	0.0510 (12)	0.0432 (12)	0.0425 (12)	0.0130 (10)	−0.0015 (9)	0.0093 (9)
N4	0.0458 (12)	0.0496 (12)	0.0444 (11)	0.0139 (9)	0.0023 (9)	0.0035 (9)
O1	0.0378 (13)	0.0342 (12)	0.0433 (12)	0.000	0.000	0.0036 (10)
C1	0.0445 (15)	0.0709 (19)	0.083 (2)	−0.0065 (14)	0.0094 (14)	0.0056 (15)
C2	0.0360 (12)	0.0457 (13)	0.0493 (13)	0.0050 (11)	−0.0013 (10)	−0.0036 (10)
C3	0.0317 (12)	0.0394 (12)	0.0401 (12)	0.0036 (10)	−0.0051 (9)	0.0018 (9)
C4	0.0390 (13)	0.0449 (14)	0.0504 (14)	0.0029 (11)	−0.0118 (10)	0.0088 (10)
C5	0.0577 (18)	0.0673 (19)	0.098 (2)	−0.0160 (15)	−0.0166 (15)	0.0370 (16)
C6	0.094 (2)	0.096 (2)	0.0436 (15)	0.0424 (19)	0.0033 (14)	−0.0021 (14)
C7	0.0452 (14)	0.0388 (12)	0.0394 (13)	0.0080 (11)	0.0003 (10)	0.0028 (9)
C8	0.0333 (12)	0.0347 (11)	0.0399 (12)	0.0011 (10)	−0.0017 (9)	0.0028 (9)
C9	0.0377 (13)	0.0449 (13)	0.0416 (12)	0.0052 (11)	0.0007 (10)	0.0048 (10)
C10	0.073 (2)	0.079 (2)	0.0487 (16)	0.0162 (16)	0.0103 (14)	0.0079 (13)

### Geometric parameters (Å, º)

**Table:**

N1—C2	1.342 (3)	C3—C8	1.474 (3)
N1—N2	1.351 (3)	C4—C5	1.497 (3)
N1—H1	0.91 (5)	C5—H5A	0.9600
N2—C4	1.342 (3)	C5—H5B	0.9600
N2—H2	0.91 (5)	C5—H5C	0.9600
N3—C7	1.331 (3)	C6—C7	1.492 (3)
N3—N4	1.357 (3)	C6—H6A	0.9600
N3—H3	0.88 (3)	C6—H6B	0.9600
N4—C9	1.339 (3)	C6—H6C	0.9600
O1—H1D	0.844 (9)	C7—C8	1.386 (3)
C1—C2	1.486 (3)	C8—C9	1.409 (3)
C1—H1A	0.9600	C9—C10	1.494 (3)
C1—H1B	0.9600	C10—H10A	0.9600
C1—H1C	0.9600	C10—H10B	0.9600
C2—C3	1.406 (3)	C10—H10C	0.9600
C3—C4	1.397 (3)		
			
C2—N1—N2	109.32 (19)	C4—C5—H5B	109.5
C2—N1—H1	134 (4)	H5A—C5—H5B	109.5
N2—N1—H1	117 (4)	C4—C5—H5C	109.5
C4—N2—N1	108.3 (2)	H5A—C5—H5C	109.5
C4—N2—H2	132 (4)	H5B—C5—H5C	109.5
N1—N2—H2	119 (4)	C7—C6—H6A	109.5
C7—N3—N4	112.30 (18)	C7—C6—H6B	109.5
C7—N3—H3	127.5 (17)	H6A—C6—H6B	109.5
N4—N3—H3	119.9 (17)	C7—C6—H6C	109.5
C9—N4—N3	105.01 (18)	H6A—C6—H6C	109.5
C2—C1—H1A	109.5	H6B—C6—H6C	109.5
C2—C1—H1B	109.5	N3—C7—C8	107.43 (19)
H1A—C1—H1B	109.5	N3—C7—C6	121.0 (2)
C2—C1—H1C	109.5	C8—C7—C6	131.5 (2)
H1A—C1—H1C	109.5	C7—C8—C9	104.47 (19)
H1B—C1—H1C	109.5	C7—C8—C3	126.7 (2)
N1—C2—C3	108.5 (2)	C9—C8—C3	128.66 (19)
N1—C2—C1	121.2 (2)	N4—C9—C8	110.78 (19)
C3—C2—C1	130.3 (2)	N4—C9—C10	120.1 (2)
C4—C3—C2	104.54 (19)	C8—C9—C10	129.1 (2)
C4—C3—C8	129.9 (2)	C9—C10—H10A	109.5
C2—C3—C8	125.59 (19)	C9—C10—H10B	109.5
N2—C4—C3	109.4 (2)	H10A—C10—H10B	109.5
N2—C4—C5	121.4 (2)	C9—C10—H10C	109.5
C3—C4—C5	129.2 (2)	H10A—C10—H10C	109.5
C4—C5—H5A	109.5	H10B—C10—H10C	109.5
			
C2—N1—N2—C4	−0.3 (3)	N4—N3—C7—C6	179.7 (2)
C7—N3—N4—C9	−0.4 (3)	N3—C7—C8—C9	−0.4 (2)
N2—N1—C2—C3	0.4 (3)	C6—C7—C8—C9	−179.4 (3)
N2—N1—C2—C1	178.0 (2)	N3—C7—C8—C3	−176.4 (2)
N1—C2—C3—C4	−0.3 (3)	C6—C7—C8—C3	4.5 (4)
C1—C2—C3—C4	−177.7 (3)	C4—C3—C8—C7	−122.9 (3)
N1—C2—C3—C8	−179.9 (2)	C2—C3—C8—C7	56.6 (3)
C1—C2—C3—C8	2.7 (4)	C4—C3—C8—C9	62.0 (3)
N1—N2—C4—C3	0.1 (3)	C2—C3—C8—C9	−118.5 (3)
N1—N2—C4—C5	179.5 (2)	N3—N4—C9—C8	0.2 (3)
C2—C3—C4—N2	0.2 (3)	N3—N4—C9—C10	179.7 (2)
C8—C3—C4—N2	179.7 (2)	C7—C8—C9—N4	0.1 (3)
C2—C3—C4—C5	−179.3 (3)	C3—C8—C9—N4	176.0 (2)
C8—C3—C4—C5	0.3 (4)	C7—C8—C9—C10	−179.3 (3)
N4—N3—C7—C8	0.5 (3)	C3—C8—C9—C10	−3.4 (4)

### Hydrogen-bond geometry (Å, º)

**Table:**

*D*—H···*A*	*D*—H	H···*A*	*D*···*A*	*D*—H···*A*
O1—H1*D*···N4	0.84 (1)	1.95 (1)	2.791 (2)	175 (2)
N3—H3···O1^i^	0.88 (3)	1.96 (3)	2.827 (2)	169 (2)
N2—H2···N2^ii^	0.91 (5)	2.23 (5)	3.015 (4)	144 (5)
N1—H1···N1^iii^	0.91 (5)	1.98 (5)	2.862 (4)	164 (5)

Symmetry codes: (i) −*y*+7/4, *x*+1/4, *z*−1/4; (ii) *y*+1/4, *x*−1/4, −*z*+7/4; (iii) −*x*+2, −*y*+3/2, *z*.
